# Estimating Case Fatality and Case Recovery Rates of COVID-19: is this the right thing to do?

**DOI:** 10.5195/cajgh.2021.489

**Published:** 2021-01-15

**Authors:** Morteza Abdullatif Khafaie, Fakher Rahim

**Affiliations:** 1 Social Determinants of Health Research Center, Ahvaz Jundishapur University of Medical Sciences, Ahvaz, Iran; 2 Thalassemia & Hemoglobinopathy Research Center, Health Research Institute, Ahvaz Jundishapur University of Medical Sciences, Ahvaz, Iran

**Keywords:** Coronavirus, COVID-19, Case fatality rates, CFRs, Case recovery rates, CRRs

## Abstract

**Introduction:**

Case fatality rates (CFRs) and case recovery rates (CRRs) are frequently used to define health consequences related to specific disease epidemics, including the COVID-19 pandemic. This study aimed to compare various methods and models for calculating CFR and CRR related to COVID-19 based on the global and national data available as of April 2020.

**Methods:**

This analytical epidemiologic study was conducted based on detailed data from 210 countries and territories worldwide in April 2020. We used three different formulas to measure CFR and CRR, considering all possible scenarios.

**Results:**

We included information for 72 countries with more than 1,000 cases of COVID-19. Overall, using first, second, and third estimation models, the CFR were 6.22%, 21.20%, and 8.67%, respectively; similarly, the CRR was estimated as 23.21%, 78.86%, 32.23%, respectively. We have shown that CFRs vary so much spatially and depend on the estimation method and timing of case reports, likely resulting in overestimation.

**Conclusions:**

Even with the more precise method of CFRs estimation, the value is overestimated. Case fatality and recovery rates should not be the only measures used to evaluate disease severity, and the better assessment measures need to be developed as indicators of countries’ performance during COVID-19 pandemic.

In late December 2019, a series of unexplained pneumonia cases were reported in Wuhan, China, which led government and researchers in China to take quick action to control its spread and start a large number of etiologic studies.^1^ On January 30, 2020, WHO declared the epidemic of the virus as a public health emergency with international concern (PHEIC).^2^ COVID-19 has spread to more than 210 countries and territories around the world, and as of December 2020, nearly 1.7 million lives have been lost.^3^ The virus spreads through droplets after infected persons cough or sneeze, which may enter the body through inhalation or contact with contaminated surfaces, and then touching the eyes, nose, and mouth.^5^ According to scientists, the average time required for symptoms to appear is 5 days, but in some cases and situations, it may take much longer, as the virus' incubation period lasts up to 14 days.^6^

Case fatality rates (CFRs) and case recovery rates (CRRs) are frequently used to define health consequences related to certain disease epidemics, as well as for the COVID-19 outbreak.^7^ CFR is the proportion of deaths due to a specified health condition compared to total infected cases.^8^ Calculations are based on the controversial assumption that all of patients were tested. COVID-related CFR might be either overestimated or underestimated depending on if calculations are based on every confirmed case or only those cases who have recovered or died. Specialists in epidemiology have proposed different scenarios for calculating CFR, each with its advantages and disadvantages.^9^-^11^ CRR is the proportion of recovered or discharged individuals with a specified health condition compared to total infected cases.^12^

The absence of reliable numbers of infected cases for the entire population could lead to inaccurate calculation of the CFR and CRR due to lack of a valid denominator. There has been an urgent need for these reported data to be openly available, so estimates of CFR and CRR can be estimated as accurately as possible. This study aimed to compare various introduced methods and models for the calculation of CFR and CRR related to COVID-19 over a time based on the recent global and national data.

## Methods

### Design and setting

This analytical epidemiologic study was conducted using detailed data from 210 countries and territories available around the world as of April 17, 2020. The current survey was approved by the Ahvaz Jundishapur University of Medical Sciences Ethical Committee.

### Source of data and procedure

We used a method that our research team recently published to retrieve data and estimate CFR and CRR.^13^ In brief, the data about total cases, total deaths, and total recovered cases, alongside total screening tests used to diagnose COVID-19, were collected from the world's most acceptable and accurate data repositories, including WHO^14^, Worldometer^4^, the Centers for Disease Control and Prevention, and the Morbidity and Mortality Weekly Report series (provided from Centers for Disease Control and Prevention)^15^, consistent with the user's guide of data sources for patient registries.^16^ The data analyses were performed between April 17–19, 2020. Data were measured and analyzed for each country, and CFR and CRR for countries with ≥1,000 cases (n=72) are presented in the main tables. Data for the remaining countries with <1,000 cases (n=138) are accessible in the supplementary tables.

### Measuring the CFR and CRR

**Figure 1.**
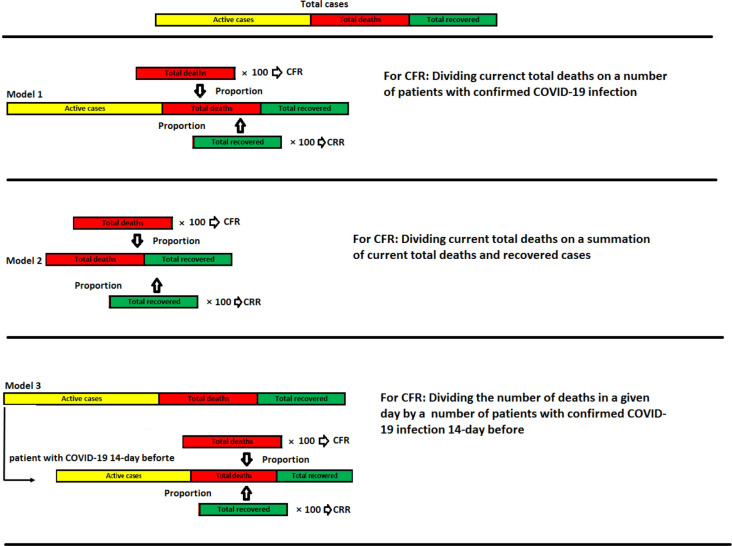
Schematic illustration of three different conceivable models for CFR and CRR calculation

Given the difficulty of estimating CFR and CRR accurately during the ongoing COVID-19 pandemic, we used three different methods to estimate CFR and CRR, considering all possible scenarios (Figure 1).

### Formula I

According to Battegay et al., we used the proportion of total deaths and recovered cases of COVID-19 disease to total cases of disease at global and national levels to estimate CFRs and CRRs, respectively.^17^

CFR = (Total deaths attributed to COVID-19/Total cases of COVID-19) * 100

CRR = (Total recovered individuals attributed to COVID-19/Total cases of COVID-19) * 100

### Formula II

Another method, proposed by Ghani et al., to estimate CFRs and CRRs is merely considering the summation of the current total deaths plus current total recovered as the denominator.^18^

CFR = Total deaths attributed to COVID-19/(deaths+recovered)

CRR = Total recovered individuals attributed to COVID-19/(deaths+recovered)

### Formula III

This formula accounts for the lag time between an individual's disease onset and death/recovery.^4^ T is the average time from emerging symptoms until the onset of death (or recovery). Since most countries had not adopted well-performing detection systems, to avoid overestimating the rates, T was considered 7 days, which is the difference of the minimum reported time between the onset of symptom to outcomes and the maximum incubation period.^19^

CFR=Deaths at day x/Total cases at day x–T

CRR=Recovered at day x/Total cases at day x–T

### Statistical analysis

Data management and calculation were conducted in Microsoft Excel, and results (CF and CR rates) were tabulated for the three standard methods of rate estimation by countries. We reported information for the 72 countries in the body of the paper with more than 1,000 cases of COVID-19 in the main paper, and the estimates of the remaining countries (n=138) were provided as supplementary tables. Overall rates for the world were also calculated.

## Results

The total number of reported cases from the beginning of the epidemic until April 17, 2020 was 1,925,179. The USA had the highest number of COVID-19 cases detected (n=578,155; 30.5% of global cases), followed by Spain and Italy with 170,099 (8.84%) and 159,516 (8.29%) cases, respectively. Table 1 shows global as well as national data on the COVID-19 health-related consequences. Global CFRs for COVID-19 estimated by first, second, and third methods were 6.22%, 21.20%, and 8.67%, respectively. Similarly, CRRs were estimated as 23.21%, 78.86%, 32.23%. The third method, which is the more precise and widely accepted method, shows that Algeria (19.17%), Belgium (18.39%), and the UK (18.38%) account for the highest CFRs. Data about all countries with confirmed cases less than 1,000 were presented in Table S1.

**Table 1.** The comparison of case fatality rate (CFR) and case recovery rate (CRR) by model between 72 different countries with at least 1,000 total cases. Data retrieved 13 September 2020.

Country	Total Recovered	Total deaths	Total cases	Active cases	Model 1		Model 2		Model 3	
					CFR1	CRR1	CFR2	CRR2	CFR3	CRR3
USA	3,950,354	198,128	6,676,601	2,528,119	2.97%	59%	4.78%	95.22%	0.85%	57.09%
India	3,702,595	78,614	4,754,356	973,147	1.65%	78%	2.08%	97.92%	0.49%	75.34%
Brazil	3,553,421	131,274	4,315,858	631,163	3.04%	82%	3.56%	96.44%	0.63%	80.79%
Russia	873,535	18,484	1,057,362	165,343	1.75%	83%	2.07%	97.93%	2.20%	78.31%
Peru	559,321	30,593	722,832	132,918	4.23%	77%	5.19%	94.81%	0.85%	61.54%
Colombia	592,820	22,734	708,964	93,410	3.21%	84%	3.69%	96.31%	2.98%	------
Mexico	467,525	70,604	663,973	125,844	10.63%	70%	13.12%	86.88%	0.44%	65.32%
South Africa	576,423	15,427	648,214	56,364	2.38%	89%	2.61%	97.39%	0.90%	77.98%
Spain	N/A	29,747	576,697	N/A	5.16%	------	------	------	0.14%	------
Argentina	409,771	11,263	546,481	125,447	2.06%	75%	2.68%	97.32%	0.85%	60.79%
Chile	404,919	11,895	432,666	15,852	2.75%	94%	2.85%	97.15%	1.06%	86.39%
Iran	344,516	23,029	399,940	32,395	5.76%	86%	6.27%	93.73%	0.68%	81.57%
France	89,059	30,910	373,911	253,942	8.27%	24%	25.76%	74.24%	1.93%	20.91%
UK	N/A	41,623	365,174	N/A	11.40%	------	------	------	1.24%	------
Bangladesh	238,271	4,702	336,044	93,071	1.40%	71%	1.94%	98.06%	0.28%	68.78%
Saudi Arabia	301,836	4,240	325,050	18,974	1.30%	93%	1.39%	98.61%	0.51%	86.03%
Pakistan	289,429	6,379	301,481	5,673	2.12%	96%	2.16%	97.84%	0.26%	93.21%
Turkey	257,731	6,999	289,635	24,905	2.42%	89%	2.64%	97.36%	0.32%	88.52%
Iraq	221,283	7,941	286,778	57,554	2.77%	77%	3.46%	96.54%	0.23%	76.62%
Italy	213,191	35,603	286,297	37,503	12.44%	74%	14.31%	85.69%	0.38%	71.24%
Germany	235,300	9,427	260,546	15,819	3.62%	90%	3.85%	96.15%	0.87%	89.92%
Philippines	187,116	4,292	257,863	66,455	1.66%	73%	2.24%	97.76%	0.06%	71.58%
Indonesia	152,458	8,650	214,746	53,638	4.03%	71%	5.37%	94.63%	0.09%	67.64%
Israel	113,496	1,103	152,722	38,123	0.72%	74%	0.96%	99.04%	0.94%	68.37%
Ukraine	68,346	3,148	151,859	80,365	2.07%	45%	4.40%	95.60%	0.36%	36.87%
Canada	120,075	9,170	136,141	6,896	6.74%	88%	7.10%	92.90%	0.01%	87.39%
Bolivia	82,796	7,297	125,982	35,889	5.79%	66%	8.10%	91.90%	0.23%	63.32%
Qatar	118,475	205	121,523	2,843	0.17%	97%	0.17%	99.83%	0.16%	91.46%
Ecuador	91,242	10,864	116,451	14,345	9.33%	78%	10.64%	89.36%	0.33%	76.72%
Kazakhstan	100,615	1,634	106,803	4,554	1.53%	94%	1.60%	98.40%	0.53%	91.90%
Dominican Republic	76,531	1,953	103,092	24,608	1.89%	74%	2.49%	97.51%	0.62%	71.19%
Romania	42,811	4,127	102,386	55,448	4.03%	42%	8.79%	91.21%	0.07%	40.11%
Panama	73,476	2,155	101,041	25,410	2.13%	73%	2.85%	97.15%	0.01%	71.66%
Egypt	83,261	5,627	100,856	11,968	5.58%	83%	6.33%	93.67%	0.08%	80.48%
Kuwait	84,404	558	94,211	9,249	0.59%	90%	0.66%	99.34%	0.26%	80.19%
Belgium	18,709	9,923	92,478	63,846	10.73%	20%	34.66%	65.34%	0.83%	18.99%
Oman	83,325	762	88,337	4,250	0.86%	94%	0.91%	99.09%	0.25%	93.35%
Sweden	N/A	5,846	86,505	N/A	6.76%	------	100.00%	------	0.49%	------
China	80,399	4,634	85,184	151	5.44%	94%	5.45%	94.55%	0.19%	92.96%
Morocco	65,867	1,553	84,435	17,015	1.84%	78%	2.30%	97.70%	0.61%	76.91%
Guatemala	70,403	2,949	81,658	8,306	3.61%	86%	4.02%	95.98%	0.25%	86.18%
Netherlands	N/A	6,253	81,012	N/A	7.72%	------	100.00%	------	0.28%	------
UAE	68,983	399	78,849	9,467	0.51%	87%	0.58%	99.42%	0.11%	80.38%
Japan	66,280	1,423	74,544	6,841	1.91%	89%	2.10%	97.90%	0.38%	87.58%
Belarus	72,547	744	73,975	684	1.01%	98%	1.02%	98.98%	0.23%	97.45%
Poland	59,725	2,182	73,650	11,743	2.96%	81%	3.52%	96.48%	0.21%	80.92%
Honduras	17,760	2,065	67,136	47,311	3.08%	26%	10.42%	89.58%	0.15%	20.56%
Ethiopia	24,493	996	63,888	38,399	1.56%	38%	3.91%	96.09%	0.40%	36.44%
Portugal	43,894	1,860	63,310	17,556	2.94%	69%	4.07%	95.93%	0.14%	67.01%
Venezuela	47,729	477	59,630	11,424	0.80%	80%	0.99%	99.01%	0.57%	77.97%
Bahrain	53,192	211	59,586	6,183	0.35%	89%	0.40%	99.60%	0.32%	85.07%
Singapore	56,699	27	57,357	631	0.05%	99%	0.05%	99.95%	0.28%	97.64%
Nigeria	44,088	1,078	56,177	11,011	1.92%	78%	2.39%	97.61%	0.35%	76.45%
Costa Rica	20,928	590	55,454	33,936	1.06%	38%	2.74%	97.26%	0.16%	32.11%
Nepal	37,524	336	53,120	15,260	0.63%	71%	0.89%	99.11%	0.81%	67.71%
Algeria	33,875	1,605	48,007	12,527	3.34%	71%	4.52%	95.48%	0.21%	68.64%
Uzbekistan	43,511	386	46,850	2,953	0.82%	93%	0.88%	99.12%	0.05%	90.71%
Switzerland	38,500	2,020	46,704	6,184	4.33%	82%	4.99%	95.01%	0.11%	76.85%
Armenia	41,605	911_	45,675	3,159	1.99%	91%	2.14%	97.86%	0.05%	89.51%
Ghana	44,342	286	45,434	806	0.63%	98%	0.64%	99.36%	0.02%	94.92%
Kyrgyzstan	40,779	1,063	44,828	2,986	2.37%	91%	2.54%	97.46%	0.62%	89.86%
Moldova	30,437	1,117	42,714	11,160	2.62%	71%	3.54%	96.46%	0.32%	69.91%
Afghanistan	31,234	1,420	38,641	5,987	3.67%	81%	4.35%	95.65%	0.03%	79.89%
Azerbaijan	35,607	559	38,172	2,006	1.46%	93%	1.55%	98.45%	0.15%	90.23%
Kenya	22,771	619	35,969	12,579	1.72%	63%	2.65%	97.35%	0.02%	60.83%
Czechia	21,205	453	35,401	13,743	1.28%	60%	2.09%	97.91%	0.25%	56.27%
Austria	26,579	754	32,696	5,363	2.31%	81%	2.76%	97.24%	0.09%	78.70%
Serbia	31,100	731	32,300	469	2.26%	96%	2.30%	97.70%	0.03%	91.26%
Ireland	23,364	1,783	30,730	5,583	5.80%	76%	7.09%	92.91%	0.19%	73.41%
Palestine	19,979	210	29,906	9,717	0.70%	67%	1.04%	98.96%	0.19%	66.07%
Paraguay	13,679	514	27,324	13,131	1.88%	50%	3.62%	96.38%	0.08%	45.81%
El Salvador	17,874	782	26,851	8,195	2.91%	67%	4.19%	95.81%	0.19%	65.70%
**World**	**20,811,464**	**924,577**	**28,943,657**	**7,207,616**	**3.19%**	**72%**	**4.25%**	**95.75%**	**0.73%**	**68.72%**

Considering the first estimation model, the highest CRRs were in China, South Korea, and Iran. Given the second estimation model, most countries such as Germany, China, Iran, Switzerland, Canada, and Austria had CRR above 90%. Based on the third estimation model, several countries, including China, Turkey, Russia, Sweden, and Peru, had CRRs higher than 90% (Table 1).

The overall lowest and highest CFR and CRR in the European continent were estimated by model 1 and model 2, respectively (Table 2). The highest CFR was observed in the European continent using models 1 and 3; model 2 highlighted the North American continent as the region with the highest CFR (Table 2). Moreover, the highest CRR was observed in Oceania in all three models (Table 2).

**Table 2.** Continental comparison of CFRs and CRRs using three various proposed estimation methods

Continents	Number of countries	Total recovered	Total deaths	Total cases	Active cases	Model 1		Model 2		Model 3	
						CFR1	CRR1	CFR2	CRR2	CFR3	CRR3
**Europe**	48	2,239,376	212,327	4,053,217	1,601,514	5.24%	55%	8.66%	55%	2.47%	47.72%
**North America**	39	4,835,653	289,160	7,950,455	2,825,642	3.64%	61%	5.64%	61%	0.52%	59.77%
**Asia**	49	6,843,427	162,543	8,485,682	1,479,712	1.92%	81%	2.32%	81%	0.17%	78.56%
**South America**	14	5,771,324	227,166	7,073,893	1,075,403	3.21%	82%	3.79%	82%	0.05%	81.19%
**Africa**	57	1,096,779	32,556	1,352,693	223,358	2.41%	81%	2.88%	81%	0.08%	80.68%
**Oceania**	7	25,940	843	29,967	3,184	2.81%	87%	3.15%	87%	0.27%	69.35%
**World**	**210**	**20,813,150**	**924,610**	**28,946,628**	**7,208,868**	**3.19%**	**72%**	**4.25%**	**72%**	**0.41%**	**70.36%**

**Figure 2.**
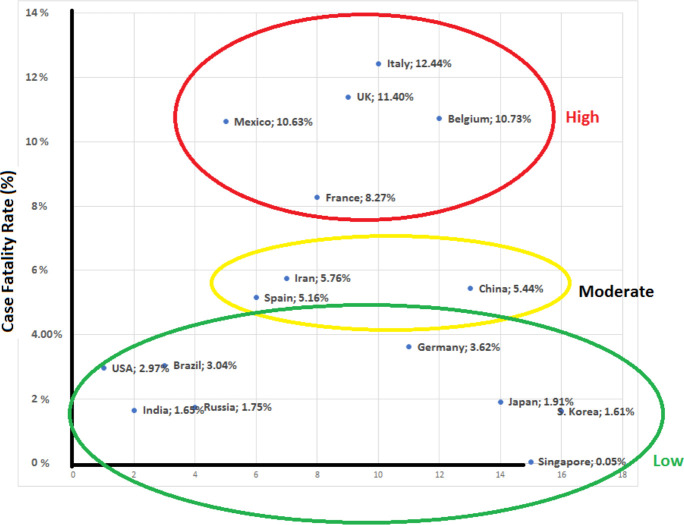
Comparison between countries with low, moderate, and high CFR

The impact of important contributing factors affecting CFR and CRR such as the country's population, GDP, number of hospital beds per 1,000 people, number of ICU beds per 100,000 people, and number of ventilators were assessed in the three different proposed models of estimation (Table S2). Comparison among countries with high, moderate, and low CFR was illustrated in Figure 2.

Though the analysis showed a statistically non-significant pattern for all variables of interest, models 1 and 2 potentially provide more accurate estimates of CFR and CRR (Table 3). The WHO reported CFR for COVID-19 as 2%^20^; other calculated values are shown based on data and available literature in countries and at the global level (Table 4).

**Table 3.** The estimated CFRs and CRRs against the county's population, GDP, number of hospital beds per 1,000 people, number of ICU beds per 100,000 people, and number of ventilators between the three different proposed models of estimation.

Variables	Model 1		Model 2		Model 3	
	*rs*	*P*	*rs*	*P*	*rs*	*P*
**Population With CFR**	0.088	0.597	-0.078	0.637	0.124	0.457
**Population With CRR**	0.098	0.556	0.078	0.637	-0.082	0.622
**GDP With CFR**	0.152	0.361	-0.029	0.859	0.266	0.106
**GDP With CRR**	0.121	0.467	0.029	0.859	0.005	0.974
**NHB With CFR**	-0.167	0.315	-0.149	0.637	0.192	0.247
**NHB With CRR**	0.124	0.457	0.149	0.371	0.121	0.468
**NIB With CFR**	0.112	0.501	0.014	0.933	0.029	0.073
**NIB With CRR**	0.122	0.462	-0.014	0.933	0.217	0.188
**Number of Ventilators With CFR**	-0.221	0.181	-0.009	0.953	0.041	0.803
**Number of Ventilators With CRR**	-0.109	0.511	0.009	0.953	-0.088	0.0595

Note: NHB: Number of Hospital Beds per 1000 people; NIB: Number of ICU Beds per 100,000 people; CFR: Case Fatality Rate; CRR: Case Recovery Rate; *r*_*s*_: Pearson Correlation Coefficient; P: P-value

**Table 4.** Reported values and methods to calculate CFR from the literature on COVID-19

Study ID (reference)	Country	Population	Method	CFR	Estimation level
Change et al, 2020 (19)	China	>30 Chinese locations and other countries/regions	Model 1 (Computational using Bayes Theorem)	3.7%	Local
Yang et al, 2020 (20)	China	205 patients with cancer and laboratory-confirmed SARS-CoV-2 infection	Model 1	Hematological malignancies: 41% Solid tumors: 3.28	Local
Turk et al., 2020 (21)	USA	474 people with intellectual and developmental disabilities (IDD)	Model 1 (CFR within 30 days)	5.1%	Local
Capalbo et al., 2020 (22)	Italy	182 patients with laboratory-confirmed SARS-CoV-2 infection	Model 2	12.1%	Local
Dongarwar and Salihu, 2020 (23)	USA	A total of 213 countries had been affected by the disease as of May 6, 2020	Model 1	Asia: 3.5 Australia: 1.4%	Global
Peng et al., 2020 (24)	China	82,836 patients with COVID-19 were confirmed in mainland China	Model 1	5.6%	Local
Abdollahi et al., 2020 (3)	Canada and USA	Using data for COVID-19 confirmed cases	Model 1 (CFR within 30 days)	Canada: 4.9% USA: 5.4%	Local
Undela and Gudi, 2020 (25)	India	2,761,121 confirmed cases	Model 1	7.0%	Global
Mi et al, 2020 (26)	China	82,735 confirmed cases	Model 1	5.7%	Local
Khafaie and Rahim, 2020 (12)	Iran	33,570 confirmed cases	Model 1 (CFR within 30 days)	3.61	Global

## Discussion

We have presented a global consequence of COVID-19 in terms of CFRs and CRRs using three different estimation methods. By April 18, 2020, deceased cases reached 119,699, according to data from Worldometer.^20^

We have shown that the CFR varies greatly geographically and even depends on the method of estimation implemented and case reports' timing. As a clear example of this, a CFR of 0.31 was estimated in Singapore and 98.82 in the UK. Even with the more precise CFR estimation method,^4^ we hypothesize that the value is still overestimated. Other factors that could contribute to varying estimations are the pandemic stage, number and types of tests performed, strategies of diagnostics, capability of the healthcare system, and the reporting system. For example, the USA had a significant increase in testing capacity, but the preliminary estimates of CFRs did not change dramatically (CFR=3.07 on March 12, 2020 vs. 4.03 on April 18, 2020).^13^ As of April 2020, most countries were testing people with severe symptoms, mainly those needing hospitalization. The important point is that it is still unclear how many cases of COVID-19 were asymptomatic, or whether similar standards for testing are being performed between countries. Cross-country comparisons cannot be reliable indicators, unless countries are comparable or important factors are adjusted for.

However, if all these possible limitations are carefully acknowledged, CFR may help better appreciate the severity of COVID-19 and required mitigation steps. Given the impossibility of accurately estimating CFR and CRR while the COVID-19 pandemic has not yet ended, using different methods to estimate CFR and CRR, considering all possible scenarios, could help us to better estimate disease severity across different countries. Some researchers prefer to use the proportion of total deaths and recovered cases of COVID-19 disease to total disease cases at global and national levels to estimate CFRs and CRRs. After the end of the pandemic, observing CFR and CRR using this method can be done, but while the pandemic is still ongoing, this method is naΪveand could be misleading.

The immune response to COVID-19 is not fully understood yet. Studies suggested the possible likelihood of relapse in recovered patients and existing models do not account for that. However, method III highly depends on the selected time period from where total cases are considered as the denominator.^18^ The estimation of CFR using method III (6.22%) is similar to the method I (8.67%). However, because all the cases have not been resolved, method III can still be assumed to be the more precise.^18^ Otherwise, we suggest merely extracting the active cases from the denominator while using method I. Undiagnosed cases are important for the disease spread, so detecting asymptomatic/undiagnosed cases is critical for the COVID-19 pandemic control. To this end, new methods based on mathematical models have been recently proposed to accurately calculate the health-related consequences of the COVID-19.^21^ One of these models is the Susceptible–Exposed–Infectious–Recovered–Dead (SEIRD) Model, which could be applied to better estimate the COVID-19 transmission rate and case fatality risk worldwide.^22^

CFR is used as a measure of disease severity and ideally, should be estimated by direct follow-up of cases and ascertainment of their outcome.^23^ We have alternatively estimated the risk in a population within a specified period by dividing the number of deaths associated with the disease by the number of cases of that disease using different methods. In this current report, we have presented risk instead of “rate” because the numerator cases were not a subset of the denominator's population. All three methods of CFR estimation have their limitations. Common limitations of the methods are the undiagnosed cases and delays in reporting data. Another limitation of this research is removing countries with a relatively small number of COVID-19 confirmed cases in the main analyses, since CFR is a flawed metric of mortality risk when the sample size is small or very limited.

CFR is commonly used to measure disease severity and is often used to predict the course or outcome of a disease. It can also be used to evaluate the effectiveness of new therapies by reducing measures and improving methods. In the COVID-19 outbreak, widespread changes in CFR estimates can be misleading, which may lead to underestimating the potential threat of COVID-19 in symptomatic patients. It is difficult to compare estimates across the countries, as different countries use different definitions and various testing strategies that may or may not include some cases. Changes in CFR may also be impacted by testing delays, dealing with delays, and differences in the quality of care or interventions at diverse stages of the disease.

Moreover, gender, ethnicity, and underlying diseases may vary by country. Cross-sectional comparisons of CFR values may be biased because the disease duration may potentially vary from country to country during the epidemic. To avoid this bias, time-adjusted estimates between the onset of symptoms and death should be recommended to compare CFRs across countries.^13^ Therefore, the estimation of CFR in response to COVID-19 pandemic disease is a high priority, but its interpretation must be done using evidence-based strategies. Though each model has its disadvantages and pitfalls, we recommend estimating CFR using corrected model I by dividing the number of deaths on a given day by the number of patients with confirmed COVID-19 infection 14 days before, based on the assumed maximum incubation period of up to 14 days.

The WHO announced that the fatality rate of the COVID-19 is 10 times higher than that of influenza, making this research timely and relevant.^14^ Due to high mortality cases around the world, accurate calculations and clear estimates of CFR for COVID-19 can inform public health interventions and policies to improve health locally and globally. CFR and CRR are not the only measures of severity of the disease, and better estimators could be explored in future research.

## Appendix

**Table S1.** The comparison of case fatality rate (CFR) and case recovery rate (CRR) between different countries (n = 210 countries and territories around the world and 2 international conveyances). Data retrieved on September 13, 2020.

Country	Total Recovered	Total deaths	Total cases	Active cases	CFR1	CRR1	CFR2	CFR2	CFR3	CFR3
USA	3,950,354	198,128	6,676,601	2,528,119	2.97%	59%	4.78%	95.22%	0.85%	57.09%
India	3,702,595	78,614	4,754,356	973,147	1.65%	78%	2.08%	97.92%	0.49%	75.34%
Brazil	3,553,421	131,274	4,315,858	631,163	3.04%	82%	3.56%	96.44%	0.63%	80.79%
Russia	873,535	18,484	1,057,362	165,343	1.75%	83%	2.07%	97.93%	2.20%	78.31%
Peru	559,321	30,593	722,832	132,918	4.23%	77%	5.19%	94.81%	0.85%	61.54%
Colombia	592,820	22,734	708,964	93,410	3.21%	84%	3.69%	96.31%	2.98%	----
Mexico	467,525	70,604	663,973	125,844	10.63%	70%	13.12%	86.88%	0.44%	65.32%
South Africa	576,423	15,427	648,214	56,364	2.38%	89%	2.61%	97.39%	0.90%	77.98%
Spain	N/A	29,747	576,697	N/A	5.16%	----	100.00%	----	0.14%	----
Argentina	409,771	11,263	546,481	125,447	2.06%	75%	2.68%	97.32%	0.85%	60.79%
Chile	404,919	11,895	432,666	15,852	2.75%	94%	2.85%	97.15%	1.06%	86.39%
Iran	344,516	23,029	399,940	32,395	5.76%	86%	6.27%	93.73%	0.68%	81.57%
France	89,059	30,910	373,911	253,942	8.27%	24%	25.76%	74.24%	1.93%	20.91%
UK	N/A	41,623	365,174	N/A	11.40%	----	100.00%	----	1.24%	----
Bangladesh	238,271	4,702	336,044	93,071	1.40%	71%	1.94%	98.06%	0.28%	68.78%
Saudi Arabia	301,836	4,240	325,050	18,974	1.30%	93%	1.39%	98.61%	0.51%	86.03%
Pakistan	289,429	6,379	301,481	5,673	2.12%	96%	2.16%	97.84%	0.26%	93.21%
Turkey	257,731	6,999	289,635	24,905	2.42%	89%	2.64%	97.36%	0.32%	88.52%
Iraq	221,283	7,941	286,778	57,554	2.77%	77%	3.46%	96.54%	0.23%	76.62%
Italy	213,191	35,603	286,297	37,503	12.44%	74%	14.31%	85.69%	0.38%	71.24%
Germany	235,300	9,427	260,546	15,819	3.62%	90%	3.85%	96.15%	0.87%	89.92%
Philippines	187,116	4,292	257,863	66,455	1.66%	73%	2.24%	97.76%	0.06%	71.58%
Indonesia	152,458	8,650	214,746	53,638	4.03%	71%	5.37%	94.63%	0.09%	67.64%
Israel	113,496	1,103	152,722	38,123	0.72%	74%	0.96%	99.04%	0.94%	68.37%
Ukraine	68,346	3,148	151,859	80,365	2.07%	45%	4.40%	95.60%	0.36%	36.87%
Canada	120,075	9,170	136,141	6,896	6.74%	88%	7.10%	92.90%	0.01%	87.39%
Bolivia	82,796	7,297	125,982	35,889	5.79%	66%	8.10%	91.90%	0.23%	63.32%
Qatar	118,475	205	121,523	2,843	0.17%	97%	0.17%	99.83%	0.16%	91.46%
Ecuador	91,242	10,864	116,451	14,345	9.33%	78%	10.64%	89.36%	0.33%	76.72%
Kazakhstan	100,615	1,634	106,803	4,554	1.53%	94%	1.60%	98.40%	0.53%	91.90%
Dominican Republic	76,531	1,953	103,092	24,608	1.89%	74%	2.49%	97.51%	0.62%	71.19%
Romania	42,811	4,127	102,386	55,448	4.03%	42%	8.79%	91.21%	0.07%	40.11%
Panama	73,476	2,155	101,041	25,410	2.13%	73%	2.85%	97.15%	0.01%	71.66%
Egypt	83,261	5,627	100,856	11,968	5.58%	83%	6.33%	93.67%	0.08%	80.48%
Kuwait	84,404	558	94,211	9,249	0.59%	90%	0.66%	99.34%	0.26%	80.19%
Belgium	18,709	9,923	92,478	63,846	10.73%	20%	34.66%	65.34%	0.83%	18.99%
Oman	83,325	762	88,337	4,250	0.86%	94%	0.91%	99.09%	0.25%	93.35%
Sweden	N/A	5,846	86,505	N/A	6.76%	----	100.00%	----	0.49%	----
China	80,399	4,634	85,184	151	5.44%	94%	5.45%	94.55%	0.19%	92.96%
Morocco	65,867	1,553	84,435	17,015	1.84%	78%	2.30%	97.70%	0.61%	76.91%
Guatemala	70,403	2,949	81,658	8,306	3.61%	86%	4.02%	95.98%	0.25%	86.18%
Netherlands	N/A	6,253	81,012	N/A	7.72%	----	100.00%	----	0.28%	----
UAE	68,983	399	78,849	9,467	0.51%	87%	0.58%	99.42%	0.11%	80.38%
Japan	66,280	1,423	74,544	6,841	1.91%	89%	2.10%	97.90%	0.38%	87.58%
Belarus	72,547	744	73,975	684	1.01%	98%	1.02%	98.98%	0.23%	97.45%
Poland	59,725	2,182	73,650	11,743	2.96%	81%	3.52%	96.48%	0.21%	80.92%
Honduras	17,760	2,065	67,136	47,311	3.08%	26%	10.42%	89.58%	0.15%	20.56%
Ethiopia	24,493	996	63,888	38,399	1.56%	38%	3.91%	96.09%	0.40%	36.44%
Portugal	43,894	1,860	63,310	17,556	2.94%	69%	4.07%	95.93%	0.14%	67.01%
Venezuela	47,729	477	59,630	11,424	0.80%	80%	0.99%	99.01%	0.57%	77.97%
Bahrain	53,192	211	59,586	6,183	0.35%	89%	0.40%	99.60%	0.32%	85.07%
Singapore	56,699	27	57,357	631	0.05%	99%	0.05%	99.95%	0.28%	97.64%
Nigeria	44,088	1,078	56,177	11,011	1.92%	78%	2.39%	97.61%	0.35%	76.45%
Costa Rica	20,928	590	55,454	33,936	1.06%	38%	2.74%	97.26%	0.16%	32.11%
Nepal	37,524	336	53,120	15,260	0.63%	71%	0.89%	99.11%	0.81%	67.71%
Algeria	33,875	1,605	48,007	12,527	3.34%	71%	4.52%	95.48%	0.21%	68.64%
Uzbekistan	43,511	386	46,850	2,953	0.82%	93%	0.88%	99.12%	0.05%	90.71%
Switzerland	38,500	2,020	46,704	6,184	4.33%	82%	4.99%	95.01%	0.11%	76.85%
Armenia	41,605	911	45,675	3,159	1.99%	91%	2.14%	97.86%	0.05%	89.51%
Ghana	44,342	286	45,434	806	0.63%	98%	0.64%	99.36%	0.02%	94.92%
Kyrgyzstan	40,779	1,063	44,828	2,986	2.37%	91%	2.54%	97.46%	0.62%	89.86%
Moldova	30,437	1,117	42,714	11,160	2.62%	71%	3.54%	96.46%	0.32%	69.91%
Afghanistan	31,234	1,420	38,641	5,987	3.67%	81%	4.35%	95.65%	0.03%	79.89%
Azerbaijan	35,607	559	38,172	2,006	1.46%	93%	1.55%	98.45%	0.15%	90.23%
Kenya	22,771	619	35,969	12,579	1.72%	63%	2.65%	97.35%	0.02%	60.83%
Czechia	21,205	453	35,401	13,743	1.28%	60%	2.09%	97.91%	0.25%	56.27%
Austria	26,579	754	32,696	5,363	2.31%	81%	2.76%	97.24%	0.09%	78.70%
Serbia	31,100	731	32,300	469	2.26%	96%	2.30%	97.70%	0.03%	91.26%
Ireland	23,364	1,783	30,730	5,583	5.80%	76%	7.09%	92.91%	0.19%	73.41%
Palestine	19,979	210	29,906	9,717	0.70%	67%	1.04%	98.96%	0.19%	66.07%
Paraguay	13,679	514	27,324	13,131	1.88%	50%	3.62%	96.38%	0.08%	45.81%
El Salvador	17,874	782	26,851	8,195	2.91%	67%	4.19%	95.81%	0.19%	65.70%
Australia	23,340	810	26,651	2,501	3.04%	88%	3.35%	96.65%	0.23%	85.10%
Lebanon	7,936	239	23,669	15,494	1.01%	34%	2.92%	97.08%	0.05%	32.87%
Bosnia and Herzegovina	15,922	690	23,138	6,526	2.98%	69%	4.15%	95.85%	0.08%	63.57%
Libya	12,100	354	22,348	9,894	1.58%	54%	2.84%	97.16%	0.18%	52.02%
S. Korea	18,226	358	22,176	3,592	1.61%	82%	1.93%	98.07%	0.37%	81.19%
Cameroon	18,837	415	20,009	757	2.07%	94%	2.16%	97.84%	0.32%	91.38%
Denmark	16,247	630	19,557	2,680	3.22%	83%	3.73%	96.27%	0.29%	80.39%
Ivory Coast	17,960	119	18,916	837	0.63%	95%	0.66%	99.34%	0.10%	92.82%
Bulgaria	12,758	717	17,891	4,416	4.01%	71%	5.32%	94.68%	0.32%	70.16%
Madagascar	14,349	210	15,737	1,178	1.33%	91%	1.44%	98.56%	0.25%	89.56%
North Macedonia	13,128	646	15,694	1,920	4.12%	84%	4.69%	95.31%	0.09%	80.47%
Senegal	10,373	295	14,237	3,569	2.07%	73%	2.77%	97.23%	0.05%	71.13%
Sudan	6,731	834	13,470	5,905	6.19%	50%	11.02%	88.98%	0.03%	44.13%
Zambia	12,007	312	13,466	1,147	2.32%	89%	2.53%	97.47%	0.01%	85.62%
Croatia	10,721	218	13,368	2,429	1.63%	80%	1.99%	98.01%	0.40%	79.47%
Greece	3,804	302	13,036	8,930	2.32%	29%	7.36%	92.64%	0.30%	27.04%
Norway	10,371	265	12,079	1,443	2.19%	86%	2.49%	97.51%	0.12%	84.63%
Hungary	4,058	633	11,825	7,134	5.35%	34%	13.49%	86.51%	0.11%	32.06%
Albania	6,494	330	11,185	4,361	2.95%	58%	4.84%	95.16%	0.36%	54.62%
DRC	9,719	262	10,385	404	2.52%	94%	2.62%	97.38%	0.27%	89.52%
Guinea	9,251	63	10,020	706	0.63%	92%	0.68%	99.32%	0.09%	89.49%
Malaysia	9,189	128	9,868	551	1.30%	93%	1.37%	98.63%	0.13%	86.58%
Namibia	5,811	98	9,604	3,695	1.02%	61%	1.66%	98.34%	0.25%	59.00%
French Guiana	9,132	63	9,521	326	0.66%	96%	0.69%	99.31%	0.30%	91.87%
Maldives	7,055	31	9,052	1,966	0.34%	78%	0.44%	99.56%	0.07%	74.77%
Tajikistan	7,782	72	9,014	1,160	0.80%	86%	0.92%	99.08%	0.09%	81.95%
Gabon	7,706	53	8,643	884	0.61%	89%	0.68%	99.32%	0.71%	88.24%
Finland	7,500	337	8,557	720	3.94%	88%	4.30%	95.70%	0.49%	82.17%
Haiti	6,120	219	8,478	2,139	2.58%	72%	3.45%	96.55%	0.18%	67.63%
Zimbabwe	5,675	224	7,508	1,609	2.98%	76%	3.80%	96.20%	0.09%	73.91%
Mauritania	6,804	161	7,274	309	2.21%	94%	2.31%	97.69%	0.56%	92.66%
Luxembourg	6,397	124	7,194	673	1.72%	89%	1.90%	98.10%	0.21%	88.24%
Tunisia	1,991	107	6,635	4,537	1.61%	30%	5.10%	94.90%	0.53%	24.70%
Montenegro	4,491	118	6,530	1,921	1.81%	69%	2.56%	97.44%	0.09%	66.39%
Malawi	3,724	177	5,678	1,777	3.12%	66%	4.54%	95.46%	0.46%	65.41%
Slovakia	3,114	38	5,453	2,301	0.70%	57%	1.21%	98.79%	0.51%	56.19%
Djibouti	5,327	61	5,394	6	1.13%	99%	1.13%	98.87%	0.07%	93.46%
Eswatini	4,188	98	5,050	764	1.94%	83%	2.29%	97.71%	0.14%	76.16%
Mozambique	2,905	35	5,040	2,100	0.69%	58%	1.19%	98.81%	0.12%	51.88%
Equatorial Guinea	4,490	83	4,996	423	1.66%	90%	1.82%	98.18%	0.00%	83.87%
Hong Kong	4,613	100	4,939	226	2.02%	93%	2.12%	97.88%	0.47%	91.11%
Congo	3,887	88	4,928	953	1.79%	79%	2.21%	97.79%	0.08%	75.95%
Nicaragua	2,913	144	4,818	1,761	2.99%	60%	4.71%	95.29%	0.15%	59.86%
CAR	1,825	62	4,749	2,862	1.31%	38%	3.29%	96.71%	0.29%	36.03%
Cabo Verde	4,104	44	4,711	563	0.93%	87%	1.06%	98.94%	0.04%	85.35%
Uganda	1,998	52	4,703	2,653	1.11%	42%	2.54%	97.46%	0.21%	36.06%
Cuba	3,878	108	4,653	667	2.32%	83%	2.71%	97.29%	0.21%	80.29%
Suriname	3,788	93	4,579	698	2.03%	83%	2.40%	97.60%	0.17%	80.78%
Rwanda	2,544	22	4,565	1,999	0.48%	56%	0.86%	99.14%	0.15%	51.59%
Jamaica	1,072	40	3,623	2,511	1.10%	30%	3.60%	96.40%	0.55%	22.74%
Slovenia	2,699	135	3,603	769	3.75%	75%	4.76%	95.24%	0.28%	73.58%
Syria	827	152	3,506	2,527	4.34%	24%	15.53%	84.47%	0.63%	22.99%
Thailand	3,312	58	3,473	103	1.67%	95%	1.72%	98.28%	0.00%	88.89%
Gambia	1,617	102	3,376	1,657	3.02%	48%	5.93%	94.07%	0.03%	47.63%
Somalia	2,791	98	3,376	487	2.90%	83%	3.39%	96.61%	0.27%	79.86%
Mayotte	2,964	40	3,374	370	1.19%	88%	1.33%	98.67%	0.00%	87.34%
Angola	1,289	132	3,335	1,914	3.96%	39%	9.29%	90.71%	0.09%	37.36%
Lithuania	2,070	86	3,296	1,140	2.61%	63%	3.99%	96.01%	0.24%	62.23%
Sri Lanka	2,983	12	3,195	200	0.38%	93%	0.40%	99.60%	0.00%	90.45%
Guadeloupe	837	24	3,080	2,219	0.78%	27%	2.79%	97.21%	0.00%	21.40%
Jordan	2,156	22	3,062	884	0.72%	70%	1.01%	98.99%	0.46%	67.90%
Aruba	1,542	18	2,994	1,434	0.60%	52%	1.15%	98.85%	0.40%	48.76%
Trinidad and Tobago	766	51	2,993	2,176	1.70%	26%	6.24%	93.76%	0.17%	25.06%
Bahamas	1,319	67	2,928	1,542	2.29%	45%	4.83%	95.17%	0.00%	40.57%
Mali	2,276	128	2,916	512	4.39%	78%	5.32%	94.68%	0.03%	73.80%
Myanmar	676	16	2,796	2,104	0.57%	24%	2.31%	97.69%	0.57%	23.28%
Réunion	1,313	14	2,723	1,396	0.51%	48%	1.06%	98.94%	0.00%	45.46%
Estonia	2,252	64	2,655	339	2.41%	85%	2.76%	97.24%	0.11%	82.94%
South Sudan	1,290	49	2,578	1,239	1.90%	50%	3.66%	96.34%	0.00%	45.42%
Guinea-Bissau	1,127	39	2,275	1,109	1.71%	50%	3.34%	96.66%	0.35%	46.95%
Malta	1,850	15	2,274	409	0.66%	81%	0.80%	99.20%	0.04%	77.53%
Botswana	546	10	2,252	1,696	0.44%	24%	1.80%	98.20%	0.27%	22.29%
Benin	1,793	40	2,242	409	1.78%	80%	2.18%	97.82%	0.04%	79.93%
Iceland	2,085	10	2,162	67	0.46%	96%	0.48%	99.52%	0.09%	93.06%
Sierra Leone	1,634	72	2,096	390	3.44%	78%	4.22%	95.78%	0.29%	75.00%
Georgia	1,363	19	2,075	693	0.92%	66%	1.37%	98.63%	0.19%	63.66%
Yemen	1,211	582	2,009	216	28.97%	60%	32.46%	67.54%	0.20%	59.78%
Guyana	1,191	54	1,812	567	2.98%	66%	4.34%	95.66%	0.17%	63.41%
New Zealand	1,676	24	1,797	97	1.34%	93%	1.41%	98.59%	0.06%	90.21%
Uruguay	1,502	45	1,780	233	2.53%	84%	2.91%	97.09%	0.62%	83.15%
Togo	1,189	37	1,555	329	2.38%	76%	3.02%	96.98%	0.39%	73.95%
Cyprus	1,281	22	1,523	220	1.44%	84%	1.69%	98.31%	0.00%	81.02%
Burkina Faso	1,127	56	1,514	331	3.70%	74%	4.73%	95.27%	0.40%	73.91%
Latvia	1,248	35	1,464	181	2.39%	85%	2.73%	97.27%	0.00%	84.43%
Belize	458	19	1,458	981	1.30%	31%	3.98%	96.02%	0.89%	29.15%
Andorra	943	53	1,344	348	3.94%	70%	5.32%	94.68%	0.60%	69.05%
Liberia	1,210	82	1,316	24	6.23%	92%	6.35%	93.65%	0.08%	90.58%
Lesotho	528	33	1,245	684	2.65%	42%	5.88%	94.12%	0.08%	41.77%
Niger	1,100	69	1,178	9	5.86%	93%	5.90%	94.10%	0.08%	92.53%
Chad	938	80	1,083	65	7.39%	87%	7.86%	92.14%	0.09%	83.56%
Vietnam	910	35	1,060	115	3.30%	86%	3.70%	96.30%	0.19%	84.15%
French Polynesia	642	2	953	309	0.21%	67%	0.31%	99.69%	0.00%	62.85%
Martinique	98	18	939	823	1.92%	10%	15.52%	84.48%	0.00%	8.73%
Sao Tome and Principe	866	15	906	25	1.66%	96%	1.70%	98.30%	0.00%	93.93%
San Marino	662	42	722	18	5.82%	92%	5.97%	94.03%	0.00%	87.26%
Diamond Princess	651	13	712	48	1.83%	91%	1.96%	98.04%	0.42%	88.76%
Turks and Caicos	270	5	641	366	0.78%	42%	1.82%	98.18%	0.00%	40.09%
Channel Islands	575	48	633	10	7.58%	91%	7.70%	92.30%	0.47%	87.05%
Sint Maarten	430	19	533	84	3.56%	81%	4.23%	95.77%	0.00%	78.80%
Tanzania	183	21	509	305	4.13%	36%	10.29%	89.71%	0.59%	35.17%
Papua New Guinea	232	5	508	271	0.98%	46%	2.11%	97.89%	0.79%	44.69%
Taiwan	475	7	498	16	1.41%	95%	1.45%	98.55%	0.40%	94.18%
Burundi	374	1	471	96	0.21%	79%	0.27%	99.73%	0.64%	77.07%
Comoros	415	7	456	34	1.54%	91%	1.66%	98.34%	0.00%	90.57%
Faeroe Islands	410		418	8	0.00%	98%	0.00%	100.00%	0.24%	98.09%
Mauritius	335	10	361	16	2.77%	93%	2.90%	97.10%	0.00%	90.03%
Eritrea	304		361	57	0.00%	84%	0.00%	100.00%	0.00%	82.27%
Isle of Man	312	24	337	1	7.12%	93%	7.14%	92.86%	0.59%	90.80%
Gibraltar	294		327	33	0.00%	90%	0.00%	100.00%	0.00%	86.24%
Mongolia	298		311	13	0.00%	96%	0.00%	100.00%	0.00%	93.57%
Cambodia	274		275	1	0.00%	100%	0.00%	100.00%	0.00%	93.45%
Saint Martin	107	6	256	143	2.34%	42%	5.31%	94.69%	0.39%	37.50%
Bhutan	159		244	85	0.00%	65%	0.00%	100.00%	0.00%	59.84%
Cayman Islands	204	1	208	3	0.48%	98%	0.49%	99.51%	0.00%	94.23%
Barbados	158	7	180	15	3.89%	88%	4.24%	95.76%	0.00%	86.67%
Bermuda	161	9	177	7	5.08%	91%	5.29%	94.71%	0.00%	82.49%
Monaco	123	1	169	45	0.59%	73%	0.81%	99.19%	0.00%	68.05%
Brunei	139	3	145	3	2.07%	96%	2.11%	97.89%	2.07%	91.03%
Curaçao	56	1	145	88	0.69%	39%	1.75%	98.25%	0.00%	31.03%
Seychelles	136		139	3	0.00%	98%	0.00%	100.00%	0.72%	94.24%
Liechtenstein	105	1	111	5	0.90%	95%	0.94%	99.06%	0.90%	90.99%
Antigua and Barbuda	91	3	95	1	3.16%	96%	3.19%	96.81%	1.05%	93.68%
British Virgin Islands	37	1	66	28	1.52%	56%	2.63%	97.37%	0.00%	39.39%
St. Vincent Grenadines	61		64	3	0.00%	95%	0.00%	100.00%	0.00%	85.94%
Macao	46		46	0	0.00%	100%	0.00%	100.00%	2.17%	82.61%
Fiji	24	2	32	6	6.25%	75%	7.69%	92.31%	3.13%	53.13%
Saint Lucia	26		27	1	0.00%	96%	0.00%	100.00%	7.41%	96.30%
Timor-Leste	25		27	2	0.00%	93%	0.00%	100.00%	0.00%	85.19%
New Caledonia	26		26	0	0.00%	100%	0.00%	100.00%	0.00%	100.00%
Caribbean Netherlands	7		25	18	0.00%	28%	0.00%	100.00%	4.00%	4.00%
Dominica	18		24	6	0.00%	75%	0.00%	100.00%	0.00%	58.33%
Grenada	24		24	0	0.00%	100%	0.00%	100.00%	4.17%	87.50%
Laos	21		23	2	0.00%	91%	0.00%	100.00%	0.00%	65.22%
St. Barth	13		21	8	0.00%	62%	0.00%	100.00%	0.00%	61.90%
Saint Kitts and Nevis	17		17	0	0.00%	100%	0.00%	100.00%	0.00%	70.59%
Greenland	14		14	0	0.00%	100%	0.00%	100.00%	0.00%	100.00%
Montserrat	11	1	13	1	7.69%	85%	8.33%	91.67%	0.00%	84.62%
Falkland Islands	13		13	0	0.00%	100%	0.00%	100.00%	0.00%	76.92%
Vatican City	12		12	0	0.00%	100%	0.00%	100.00%	0.00%	100.00%
Saint Pierre Miquelon	5		11	6	0.00%	45%	0.00%	100.00%	0.00%	36.36%
Western Sahara	8	1	10	1	10.00%	80%	11.11%	88.89%	0.00%	80.00%
MS Zaandam		2	9	7	22.22%	0%	100.00%	0.00%	0.00%	0.00%
Anguilla	3		3	0	0.00%	100%	0.00%	100.00%	0.00%	100.00%
**Total**	**20,811,464**	**924,577**	**28,943,657**	**7,207,616**	**3.19%**	**72%**	**4.25%**	**95.75%**	**0.73%**	**68.72%**

**Table S2.** The estimated CFRs and CRRs for each included country (n=38) against the county's population, GDP, number of hospital beds per 1,000 people, number of ICU beds per 100,000 people, and number of ventilators between the three different proposed models of estimation.

Country	Total Recovered	Total deaths	Total cases	Active cases	Population (Million)	GDP (Trillion)	hospital beds per 1,000 people	ICU Beds per 100,000 people	Number of Ventilators	Model 1		Model 2		Model 3	
										CFR1	CRR1	CFR2	CRR2	CFR3	CRR3
**USA**	36,948	23,644	587,155	526,563	327.2	19.39	2.77	34.7	177,000	4.03	6.29	39.02	60.98	6.96	12.18
**Spain**	64,727	17,756	170,099	87,616	46.66	1.311	2.97	9.7	NR	10.44	38.05	21.53	78.47	12.37	44.83
**Italy**	35,435	20,465	159,516	103,616	60.48	1.935	3.18	12.5	3,000	12.83	22.21	36.61	63.39	14.85	28.73
**France**	27,718	14,967	136,779	94,094	66.99	2.583	5.98	11.6	30,000	10.94	20.26	35.06	64.94	14.57	27.14
**Germany**	64,300	3,194	130,072	62,578	82.79	3.677	8.00	29.2	25,000	2.46	49.43	4.73	95.27	3.55	66.79
**UK**	135	11,329	88,621	76,948	66.44	2.622	2.54	6.6	8,175	12.78	0.15	98.82	1.18	18.35	-----
**China**	77,738	3,341	82,249	1,170	1,386	12.24	4.34	3.6	NR	4.06	94.52	4.12	95.88	5.64	93.80
**Iran**	45,983	4,585	73,303	22,735	81.16	0.4395	1.5	4.8	NR	6.25	62.73	9.07	90.93	2.64	14.58
**Turkey**	3,957	1,296	61,049	55,796	80.81	0.8511	2.81	47.1	17,000	2.12	6.48	24.67	75.33	8.83	98.30
**Belgium**	6,707	3,903	30,589	19,979	11.4	0.4927	5.76	15.9	NR	12.76	21.93	36.79	63.21	18.39	28.16
**Netherlands**	250	2,823	26,551	23,478	17.18	0.8262	3.32	6.4	NR	10.63	0.94	91.86	8.14	9.27	54.82
**Switzerland**	13,700	1,138	25,688	10,850	8.57	0.6789	4.53	11.0	NR	4.43	53.33	7.67	92.33	1.23	12.03
**Canada**	7,756	780	25,680	17,144	37.59	1.653	2.52	13.5	NR	3.04	30.20	9.14	90.86	6.03	45.96
**Brazil**	173	1,355	23,723	22,195	209.3	2.056	2.3	NR	NR	5.71	0.73	88.68	11.32	16.13	1.12
**Russia**	1,470	148	18,328	16,710	144.5	1.578	8.05	8.3	40,000	0.81	8.02	9.15	90.85	8.25	103.11
**Portugal**	277	535	16,934	16,122	10.29	0.2176	3.39	4.2	1,400	3.16	1.64	65.89	34.11	4.36	3.87
**Austria**	7,343	368	14,041	6,330	24.6	1.323	3.84	9.1	1,314	2.62	52.30	4.77	95.23	3.74	17.66
**Israel**	1,855	116	11,586	9,615	8.712	0.3509	3.02	NR	NR	1.00	16.01	5.89	94.11	5.12	0.69
**Sweden**	381	919	10,948	9,648	10.12	0.538	2.22	5.8	NR	8.39	3.48	70.69	29.31	4.20	96.93
**Ireland**	25	365	10,647	10,257	4.83	0.3337	2.96	6.5	NR	3.43	0.23	93.59	6.41	3.32	63.76
**S. Korea**	7,534	222	10,564	2,808	51.4	1.531	12.27	10.6	9,795	2.10	71.32	2.86	97.14	15.65	5.70
**India**	1,181	358	10,453	8,914	1,339	2.597	0.53	5.2	40,000	3.42	11.30	23.26	76.74	1.78	37.54
**Peru**	2,642	216	9,784	6,926	32.17	0.2114	1.6	NR	NR	2.21	27.00	7.56	92.44	3.11	96.101
**Japan**	799	143	7,645	6,703	126.8	4.872	13.05	7.3	32,586	1.87	10.45	15.18	84.82	2.97	14.32
**Ecuador**	597	355	7,529	6,577	16.62	0.1031	1.50	NR	NR	4.72	7.93	37.29	62.71	1.71	54.75
**Chile**	2,367	82	7,525	5,076	18.05	0.2771	2.2	2.11	NR	1.09	31.46	3.35	96.65	6.32	13.97
**Poland**	487	245	6,934	6,202	37.98	0.5245	6.62	6.9	10,100	3.53	7.02	33.47	66.53	5.20	14.70
**Romania**	914	331	6,633	5,388	19.53	0.2118	6.3	21.4	NR	4.99	13.78	26.59	73.41	6.45	26.51
**Norway**	32	134	6,605	6,439	5.368	0.3988	3.6	8	800	2.03	0.48	80.72	19.28	1.46	21.02
**Australia**	3,494	61	6,394	2,839	24.6	1.323	3.84	9.1	1,314	0.95	54.64	1.72	98.28	2.27	29.08
**Denmark**	2,235	285	6,318	3,798	5.603	0.3249	2.61	6.7	NR	4.51	35.38	11.31	88.69	10.53	42.55
**Czech Republic**	519	143	6,059	5,397	10.65	0.2157	6.63	11.6	3,529	2.36	8.57	21.60	78.40	5.78	64.21
**Pakistan**	1,097	96	5,707	4,514	197	0.305	0.6	NR	34,000	1.68	19.22	8.05	91.95	3.14	0.61
**Mexico**	1,964	332	5,014	2,718	129.2	1.15	1.38	1.2	2,050	6.62	39.17	14.46	85.54	3.87	26.20
**Saudi Arabia**	805	65	4,934	4,064	32.94	0.6838	2.7	NR	NR	1.32	16.32	7.47	92.53	1.50	89.40
**Philippines**	242	315	4,932	4,375	104.9	0.3136	1.0	NR	NR	6.39	4.91	56.55	43.45	0.80	25.56
**Malaysia**	2,276	77	4,817	2,464	31.62	0.3145	1.9	NR	NR	1.60	47.25	3.27	96.73	11.99	14.14
**Indonesia**	380	399	4,557	3,778	264	1.016	1.2	NR	NR	8.76	8.34	51.22	48.78	9.36	12.17
**World**	**445,023**	**119,699**	**1,925,179**	**1,360,457**	**----**	**----**	**----**	**----**	**----**	**6.22**	**23.12**	**21.20**	**78.80**	**8.67**	**32.23**
